# Supplementary material for: Knowledge, attitudes and practices regarding neonatal jaundice among caregivers in a tertiary health facility in Ghana
Source: PLoS One. 2021 Jun 4;16(6):e0251846. doi: 10.1371/journal.pone.0251846 (PMC8177863; doi:10.1371/journal.pone.0251846)
Supplement: Attachment — Submitted filename: Reviewer plosone Nov 2020.docx [file pone.0251846.s002.docx]

Reviewer:

Plos one

Title: Determinants of Neonatal Jaundice Knowledge, Attitudes and Practice Among Caregivers in a Tertiary Health Facility in Ghana: Implications for Neonatal Care policy Reforms and Reducing Neonatal Mortality in a Low Resource Setting.

Date: 26/11/2020

**General comment:**

Thank you for allowing me to review this article by Salia, et al. The manuscript is about neonatal jaundice a common condition affecting newborns which can lead to disability and sometimes mortality. Generally, the manyscript reads well and is of relevance to those with an interest in the public health aspect of newborn care. However there are several abbreviations such as Neonatal Jaundice, neonatal mortality and postnatal which are simple words or phrases, that can be obtrusive to reading and a few other items that need to be corrected prior to publication. I have made some suggestions below:

**Major corrections**

- Page 1: Title: Suggest revision of title to: Knowledge, Attitudes and Practices Regarding Neonatal Jaundice Among Caregivers in a Tertiary Health Facility in Ghana.
- This was what was done. The study is a small study and finding are not so different from what is already known or so novel to warrant a reform in policy.
- Page 6 Sample size and sampling determination (change determination to methods) - it is not clear what sampling method was actually applied. While is says in this section that systematic sampling was done, on Page 8 line 10 it says ...caregivers were conveniently selected – this is in contrast with the assertion on page 6 that systematic sampling was used. Please clarify.
- Page 6 Line 12 and 13: was selection of the first skip interval done by balloting or other random sampling method? How were the patients arranged ina row? Were they arranged according to time of arrival as one would imagine would be the same as the attendance records or in another way? This needs to be specified.
- Page 2 abstract line 12 under methods: It does not say where the study was done and what data was collected.

**Minor essential corrections**

- **Abstract**
- Page 2 line 23: suggest rephrase to “good attitude about neonatal jaundice” rather than “...attitude in jaundice”.
- Page 2 line 29: suggest rephrase to “,,, will help reduce disability and deaths from neonatal jaundice.”

**Introduction**

- Page 3 line 14: it will be useful to also capture global targets (SDG)
- Page 3 lines 9-11. Although neonatal jaundice affects a number of babies, many of them recover. A few may die from kernicterus or bilirubin encephalopathy however underlying causes such as neonatal sepsis are more often the cause of neonatal death in babies with jaundice. Nonetheless neonatal jaundice is a major cause of disability i.e. cerebral palsy and emphasis of this role is lacking. Thus need to rephrase to capture this.
- Page 4 line 9: should read “...as a means of treatment”
- Page 4 line 10 change “generations back” to “previous generations”
- Page 4 line18 suggest rephrasing to read...serve as useful information

**Methods**

- Page 5 ethical approval: were the benefits and risks also explained to them?
- **Study area:** Information on the delivery rate, presence of a newborn unit, its capacity, admission and neonatal mortality rates will be useful.
- Page 5 line 17: ...the health facility serves as a major facility for neighbouring Togo residents – needs to be put in the right perspective as unintentional.
- Page 5 line 17 bed capacity” remove approximately- be specific
- Page 6 study design – reads better without the abbreviations. i.e. if they are written in full -. KAP and NJ
- Page 6 line 20 - 21 Inclusion criteria- need to include the recruitment period
- Page 7 line 1: rephrase to “...not the parents of the baby” rather than “...not the real father or mother”
- Page 7 line 7 - no mention of northern tribes – they form a significant proportion of the Ghanaian population, were they few?
- Page 7 line 12: Adequacy of antenatal visits is now pegged at 8 or more visits but 4 or more can still be used as information on this is more readily available for comparison. Need to mention
- Page 7 line 15 -17. Add - these are captured in more details in the results section .
- Page 8 line 17: “...further explanation” seems a more appropriate to understanding
- Page 9 line 4 comment of significance of these coefficients.

Results

- Page 10. Table 1 title: choose caregivers or participants
- Page 13: frequent ANC attendance alone will not prevent jaundice unless it provides education on prevention and recognition. Sentence should reflect this.
- Page 13 Lines 4 and 5 only a few identified traditional practices as being wrong should be reflected in the discussion and conclusion
- Page 10. Table 1 an unusually large number of caregivers with tertiary education needs an explanation and additional information on catchment area for hospital or was therea selection before the sampling method was applied
- Page 13 Attitude questions seem like knowledge questions.
- Page 13 last but one sentence spelling of colour –color
- Page 17 last line & Table 7 suggests - factors associated rather than determinants Having one child was associated p-0.009 (with) ones practices, but not explained or discussed.
- Conclusion poor knowledge regarding traditional treatments not mentioned
- Discussion
- Page 20 line 22: Is it possible that the reason for the disparity between your finding and Amegan-Aho et al‘s is because there were several caregivers with tertiary education in this study?
- Page 21 line 6 suggest “...though in the minority...”
- Page 21 line 8 suggest “... poor knowledge of (omit “a”)”
- Page 21 line 14 suggest “... knowledge of... rather than “in”.
- Page 21 last line: what is meant by “positive” knowledge?
- Page 22 last but 3 line suggest removal of “will” to read who “offer”

Discretionary corrections

- Page 4 line 7 suggest revising “where” to read “because”.
- Page 8 line 11: questions asked in the questionnaire –suggest rephrase
- Page 20 Line 12: suggest revising “...where majority to “in which majority”
- Page 21 line 17 suggest removal of “pre” before suggested
