# Supplementary material for: Knowledge, attitudes and practices regarding neonatal jaundice among caregivers in a tertiary health facility in Ghana
Source: PLoS One. 2021 Jun 4;16(6):e0251846. doi: 10.1371/journal.pone.0251846 (PMC8177863; doi:10.1371/journal.pone.0251846)
Supplement: Attachment — Submitted filename: Response to Reviewers.docx [file pone.0251846.s004.docx]

**Manuscript title: Knowledge, Attitudes and Practices Regarding Neonatal Jaundice Among Caregivers in a Tertiary Health Facility in Ghana**

*Solomon Mohammed Salia^1^, Agani Afaya^1,2^, Abubakari Wuni^3^, Martin Ayanoore^4^, **Emmanuel Salia^5^**, Doreen Dzidzor Kporvi^1^, Peter Adatara^1^, Robert Kaba Alhassan^6^, **Vida Nyagre Yakong^7^**, Sean Augustine Eduah-Quansah^1^, Shine Seyram Quarshie^1^, Eric Kwame Dey^1^, Dominic Amoah Akolga^1^

Dear editor,

We are very happy that you are giving us another chance to shape our manuscript to the required journal’s standard for publication. We would like to thank the reviewers for the thorough review and insightful comments on our manuscript. The comments though minor are very useful and we have responded to them to the best of our knowledge. We acknowledge that the comments have no doubt helped improve the quality of our manuscript.

We have therefore provided further details by showing point-by-point feedback on how each of the comments by reviewer # 2 was addressed. For easy identification of our responses, the reviewers’ comments have been repeated while the Authors’ responses appear in **BOLD** text in the main manuscript.

Also, reference to my mail dated 11^th^ January 2021 requesting to include names of two authors who duly participated in our study and with subsequent mail from Noelle Gibbs, on the 12^th^ January 2021 informing us to include the same names should we receive another revised decision, I wish to include (Emmanuel Salia and Vida Nyagre Yakong) as part of the authors.

**Reviewer’s comments**

Abstract

**Reviewer’s comments**

Page 2 line 47: suggest changing “...of neonatal jaundice...” to “...regarding neonatal jaundice...”

**Authors response**

Page 2 line 50-51: the phrase “of neonatal jaundice was changed to regarding neonatal jaundice.

**Reviewer’s comments**
Page 2 line 52-53: suggest revision: “...sampling strategy. Quantitative data was collected using a questionnaire and analysed with STATA....

**Authors response**

Page 2 line 55-56: the method section of the abstract was revised to include “Quantitative data was collected using a questionnaire and analyzed with STATA”

**Reviewer’s comments**
Page 2 line 54: the phrase “...neonatal jaundice knowledge...” does not seem right, kindly revise.

**Authors response**

Page 2 line 57-58: the manuscript was revised where “neonatal jaundice knowledge” was changed to “caregivers’ knowledge regarding neonatal jaundice”

**Reviewer’s comments**
Page 2 line 57 sentence beginning with “More than 50% (54.5%) and (52.5%) of caregivers...” is unclear, kindly revise

**Authors response**

Page 2 line 60: the manuscript was revised and the sentence “more than 50% (54.5%) and (52.5%)” was replaced with “most…”

**Reviewer’s comments**
Page 2 line 59: suggest revising “....knowledge in...” to “knowledge about...”

**Authors response**

Page 2 line 62: the phrase “knowledge in” was replaced with “knowledge about” in the revised manuscript.

**Reviewer’s comments**
Conclusion – needs to be more specific and focused on findings.
**Authors response**

Page 2 line 67-72: the conclusion aspect of the manuscript has been revised to reflect the reviewer’s comment.

Introduction

**Reviewer’s comment**
Page 3 line 76-77: “Jaundice ...manifestation of a disease.” kindly add a reference.

**Authors response**

Page 3 line 80: the manuscript was revised to remove the phrase “Jaundice per se is not a disease condition, but a manifestation of a disease”

**Reviewer’s comment**

Page 3 line 99: reference 12: Reference from Graphic should be changed to a more appropriate source of data on prevalence of neonatal jaundice in Ghana.

**Authors response**

Page 31 line 607-608: this reference was revised in the reference section of the manuscript

**Reviewer’s comment**

Page 3 line 101 suggest changing revealed to reported
**Authors response**

Page 3 line 106: the word “revealed” was revised to “reported” in the main manuscript

Materials and methods

**Reviewer’s comment**
Page 6 line146: the word reinforced is unclear in this context.

**Authors response**

Page 6 line 152: the word “reinforced” was removed and the sentence was revised in the main manuscript.

**Reviewer’s comment**
Page 6 line 153-154: invasive procedures are not the only sources of risk or discomfort so suggest you remove or revise the reference. The inconvenience of additional waiting to complete the questionnaire, though minimal may be seen as a source of discomfort to some.

**Authors response**

Page 6 line 158-159: the manuscript was revised to reflect the comment.

**Reviewer’s comment**
Page 6 line 157: study area you have described the neonatal unit however it is still not clear how neonatal services are delivered at the hospital as you have not described the postnatal ward in adequate detail.

**Authors response**

Page 7 line 171-175: the manuscript was revised to include a description of the postnatal unit of the Ho teaching hospital.

**Reviewer’s comment**

Page 7 line 172: remove capitalization

**Authors response**

Page 7 line 182: the manuscript was revised in the study design to include knowledge, attitude and practice

**Reviewer’s comment**
Page 7 line 175: Change methods to determination to meet journal specification

**Authors response**

Page 7 line 185: sampling method changed to sampling determination

**Reviewer’s comment**
Page 7 line 184: sentence “Balloting was done...”. needs to be re-sequenced as the skip interval is usually determined before the starting point.
**Authors response**

Page 7 and 8 line 196: the manuscript was revised to reflect the reviewer’s comment.

**Reviewer’s comment**

Page 9 line 208 to 2010: suggest revision- Although WHO has changed the minimum recommended antenatal visits from 4 to 8 visits, in this study we studied an attendance of 4 visits.[reference] No need to mention availability of information for comparison..

**Authors response**

Page 9 line 217-219: the manuscript was revised in the study variables section and references (WHO, 2016) and (Afaya et al., 2020) included.

**Reviewer’s comment**

Page 9 line 218-219 remove capitalization

**Authors response**

Page 9 line 227-228: the manuscript was revised to remove capitalization of knowledge, attitude and practice.

**Reviewer’s comment**
Page 10 line 238 remove” For”

**Authors response**

Page 10 line 247: the word “For” was removed in the revised manuscript

Results

**Reviewer’s comment**
Format tables to fit with journals recommendations and also provide information on Total respondents

**Authors response**

Tables were formatted to journal’s specification

**Reviewer’s comment**
Table1: Title - change caregiver to caregivers; Characteristics - Education received on neonatal jaundice and Child with a diagnosis of neonatal jaundice reads better (page 14)

**Authors response**

Table 1 in the manuscript was revised as per the reviewer’s comment. See table 1 for reference.

**Reviewer’s comment**
Table 2: title- suggest - Caregivers knowledge about neonatal jaundice; Prevention of neonatal jaundice: Neonatal jaundice is a common problem on newborns...so there is no need to prevent it reads better

**Authors response**

Table 2 of the manuscript was revised to reflect the reviewer’s comment.

**Reviewer’s comment**
Table 5 and 6; more information needed on the logistic regression model. What aspects of the knowledge, attitudes, beliefs and practices and were examined?

**Authors response**

We examined for those that had good knowledge and attitude regarding neonatal jaundice

**Reviewer’s comment**
Page 19 line 354. There is no decimal place in the p-value.

**Authors response**

Page 19 line 358: the manuscript was revised to include a decimal place in the p-value as p=0.049

**Reviewer’s comment**
Conclusion: Does not provide a succinct summary of some of the good practices and gaps in knowledge.

**Authors response**

Page 26 line 483-488: the conclusion section of the manuscript was revised to include good practices and gaps in knowledge.

**Reviewer’s comment**
Lines 488-480 no need to bring in percentages.
**Authors response**

Page 26 line 489-492: the manuscript was revised to remove all percentages.

Discretionary corrections

**Reviewer’s comment**
Page 2 line 42: suggest removing word “trends”

**Authors response**

Page 2 line 45: the word “trends” was removed in the revised manuscript

**Reviewer’s comment**
Page 8 line 198; suggest removing “thus”

**Authors response**

Page 8 line 206: the word “thus” was removed.

**Reviewer’s comment**
References - Need to examine references again. Inconsistency in formatting noted,; date of accessing online publication omitted in some instances

**Authors response**

Page 31-33: References section of the manuscript was assessed and inconsistencies corrected.
