# Supplementary material for: Knowledge, attitudes and practices regarding neonatal jaundice among caregivers in a tertiary health facility in Ghana
Source: PLoS One. 2021 Jun 4;16(6):e0251846. doi: 10.1371/journal.pone.0251846 (PMC8177863; doi:10.1371/journal.pone.0251846)
Supplement: Attachment — Submitted filename: Response to Reviewers.docx [file pone.0251846.s005.docx]

**Manuscript title: Knowledge, Attitudes and Practices Regarding Neonatal Jaundice Among Caregivers in a Tertiary Health Facility in Ghana**

*Solomon Mohammed Salia^1^, Agani Afaya^1,2^, Abubakari Wuni^3^, Martin Ayanoore^4^, Emmanuel Salia^5^, Doreen Dzidzor Kporvi^1^, Peter Adatara^1^, Robert Kaba Alhassan^6^, Vida Nyagre Yakong^7^, Sean Augustine Eduah-Quansah^1^, Shine Seyram Quarshie^1^, Eric Kwame Dey^1^, Dominic Amoah Akolga^1^

Dear editor,

We are very happy that you are giving us another chance to shape our manuscript to the required journal’s standard for publication. We would like to thank the reviewers for the thorough review and insightful comments on our manuscript. The comments though minor are very useful and we have responded to them to the best of our knowledge. We acknowledge that the comments have no doubt helped improve the quality of our manuscript.

We have therefore provided further details by showing point-by-point feedback on how each of the comments was addressed. For easy identification of our responses, the comments have been repeated while the Authors’ responses appear in **BOLD** text in the main manuscript.

**Editor’s Comments**

Abstract - Results

DELETE: ‘Most of the caregivers demonstrated poor knowledge (54.5%) and attitude (52.5%) while 58.9% had good practices regarding neonatal jaundice’.

REPLACE WITH:  ‘Less than half of the caregivers demonstrated good knowledge (45.5%) and attitude (47.5%) but 58.9% had good practices regarding neonatal jaundice’.

**Authors Response**

Page 2 line 60-61: The results section of the abstract was deleted and replaced with the suggested statement by the editor which adds more meaning to the results.

**Editor’s Comment**

2. Abstract Conclusion:

DELETE: ‘Most of the caregivers demonstrated poor knowledge and attitude about jaundice while the majority demonstrated good practice. Healthcare professionals need to intensify education regarding jaundice among caregivers in low resource settings throughout antenatal and postnatal periods. Good knowledge may possibly lead to positive attitudes towards jaundice. Overall, improved knowledge and attitude improve maternal health-seeking behaviours which may help to reduce disabilities and deaths from neonatal jaundice’.

REPLACE WITH: 'Less than two thirds of the caregivers demonstrated good practice with limited knowledge and poor attitude. Efforts to promote well informed and improved caregivers’ attitude will advance positive maternal health-seeking behaviour and reduce disabilities and death through early detection and intervention of infants with neonatal jaundice. Public awareness and education about neonatal jaundice especially among caregivers in the private sector should also be intensified'.

**Authors Response**

Page 2 line 68-73: The authors have deleted the conclusion section of the abstract and have replaced it with the editor’s suggested phrase. This has made the understanding of the conclusion clearer.

**Editor’s Comment**

**3.** Reference 12 is incomplete without a URL.

**Authors Response**

Reference to the second revision, the reviewer requested that we change the graphic Online source to a more appropriate reference. However, the authors have searched and have realized that the information used by graphic online was from the Ghana Health Service (GHS), but this information from the GHS was not published as at the time the authors used the information, and therefore, did not have a URL.

Page 31 line 610-612: The authors have revised the reference and have added the URL
